# Optimal Extent of Neck Dissection for a Head and Neck Lymph Node Metastasis from a Remote Primary Site

**DOI:** 10.3390/jcm13030661

**Published:** 2024-01-23

**Authors:** Han Wool John Sung, Hyo One Son, Da Beom Heo, Ho-Ryun Won, Bon Seok Koo, Jae Won Chang

**Affiliations:** Department of Otolaryngology-Head and Neck Surgery, Chungnam National University College of Medicine, Daejeon 35015, Republic of Korea; johnsungg@gmail.com (H.W.J.S.); 20220245@cnuh.co.kr (H.O.S.); gjekqja@cnuh.co.kr (D.B.H.); hryun83@cnuh.co.kr (H.-R.W.); bskoo515@cnuh.co.kr (B.S.K.)

**Keywords:** cervical lymph node metastasis, remote primary, occult metastasis, optimal neck dissection extent, survival

## Abstract

Background: Despite its rarity and limited documentation, therapeutic neck dissection (ND) for cervical lymph node (LN) metastases from distant primary sites is increasingly practiced, potentially enhancing survival rates. However, the optimal ND extent remains unclear. This study aimed to determine the safety of excluding upper neck levels from ND. Methods: We retrospectively analyzed 25 patients who underwent ND for cervical LN metastases from remote primary tumors between 2015 and 2021 (12 with primary lung tumors, four with ovary, three with mammary gland, three with esophagus, two with thymus, and one with colon). Results: Assessing clinical characteristics and occult metastasis rates, we observed LN metastases predominantly at levels III and IV. Occult metastases occurred in 14 out of 25 patients, primarily at neck levels III and IV (55.0% and 50.0%, respectively). The five-year disease-specific survival rate for all patients was 44.3%. While no statistically significant impact of occult metastasis on prognosis was confirmed, an association between the postoperative LN ratio and poor prognosis was revealed. Conclusions: Our findings suggest that prophylactic NDs at levels I, II, and Va may not be essential for managing cervical LN metastases from remote primary malignancies. This could lead to a more tailored and less invasive therapeutic strategy.

## 1. Introduction

Approximately 1% of all cases of head and neck malignancies are caused by metastases from remote primary sites. The most frequent sites of origin include the breast, lungs, gastrointestinal tract, genitourinary tract, and rarely the central nervous system [1]. Cervical lymph node (LN) metastases from a remote primary malignancy are typically discovered at an advanced stage, resulting in a poor prognosis with limited surgical management [1].

Recent advancements in nonsurgical cancer treatments, such as targeted therapy, gene therapy, phytochemicals, and immune checkpoint inhibitors, have expanded the scope of surgical interventions aimed at reducing tumor volume and potentially enhancing survival rates [2]. This has led to the increased availability of surgical options for reducing tumor volume, which may ultimately improve survival rates. In patients with resectable cervical metastases, ND may be a viable option as a definitive therapeutic approach in selective cases if other lesions, including those at the primary site, are well managed. The recent literature suggests that ND for cervical LN metastases from remote primary malignancies may improve both quality of life and survival [3].

For head and neck squamous cell cancer with clinically positive LNs (c+Ns), the standard surgical intervention is modified radical ND (mRND), which effectively addresses potential LN involvement, including occult metastases [4]. However, mRND carries increased risks of postoperative complications, such as major vessel bleeding, chyle leakage, pneumothorax, and nerve injuries, including spinal accessory, phrenic, and marginal mandibular branches of the facial nerve, as well as cervical and brachial plexus damage [5]. Consequently, a shift towards selective ND (SND) has occurred in cases of cN1 and certain cN2 neck diseases, as SND can effectively reduce postoperative complications [6]. This approach is more conservative and targets only specific neck levels where metastases are more likely, thereby minimizing morbidity. However, in the context of cervical LN metastases from remote primary malignancies, the focus of this study, the infrequency of the cases and the heterogeneity of primary tumors have led to a paucity of data and less established optimal management [1,7]. Given this rarity, the standardization of ND procedures, particularly regarding the extent of dissection necessary for optimal patient outcomes, remains underexplored [3].

Therefore, this study aimed to clarify the incidence and risk factors of occult cervical LN metastasis from remote primary tumors and to determine the optimal extent of ND. Our findings are expected to significantly influence the management of patients with cervical LN metastases, guiding surgical planning and execution while minimizing the morbidity associated with extensive ND.

## 2. Materials and Methods

### 2.1. Ethical Considerations

The Institutional Review Board and Research Ethics Committee of Chungnam National University Hospital (Daejeon, Republic of Korea) approved this study, and the requirement for informed consent was waived because of its retrospective nature. This waiver is in compliance with institutional guidelines and ethical standards for retrospective medical research, where patient consent can be waived for studies involving minimal risk and using previously collected, de-identified data.

### 2.2. Subjects

Between March 2015 and March 2021, we performed 25 NDs to manage metachronous or isolated persistent cervical LN metastases. The study included patients who met the following criteria: (1) clinically (using fluorodeoxyglucose positron emission tomography–computed tomography (FDG-PET/CT)) and pathologically (through fine needle aspiration cytology and core needle biopsy) confirmed cervical LN metastases originating from distant primary malignancies (the pathology of the cervical LN metastasis had to be consistent with that of the primary disease); (2) patients who had completed comprehensive treatment for their primary cancer, including surgery, chemotherapy, and/or radiotherapy, and currently had no evidence of remaining lesions other than in the cervical neck. We excluded patients who (1) had cervical LN metastases without a confirmed distant primary malignancy, (2) had not completed treatment for their primary malignancy before undergoing ND, or (3) had other simultaneous primary tumors or metastatic tumors.

A multidisciplinary tumor board decided whether to perform surgery after comprehensively considering the current disease status, patient performance, and future treatment plans. Data from these patients were retrospectively investigated. The patients comprised 13 males and 12 females, with an age range of 40–74 years (mean age: 57.8 years) (Table 1). 

The primary sites were 12 lungs, four ovaries, three mammary glands, three esophagus, two thymus, and one colon. In all cases, treatment of the primary site was completed, and chemotherapy and/or radiotherapy were continued before performing ND. One patient who underwent lung cancer treatment (case 13) had recurrence in the posterior pharyngeal wall and underwent transoral robotic surgery after non-small cell lung cancer treatment and ND due to recurrence in the cervical LN 14 months after surgery. The laterality of the ND was left in 18 patients and right in 7. The histology of the cases, previous treatments, and adjuvant therapies after ND are shown in Table 1. The adjuvant therapies were determined by multidisciplinary tumor boards, with the aim of providing the most optimal and tailored treatment for each patient. This decision-making process involved careful consideration of various factors, including the nature of the primary disease, the patient’s overall health condition, and the nodal basin status. The latter encompasses factors such as the presence of occult metastasis, extranodal extension, and tumor burden, including multiple or large lymph node metastases and the lymph node ratio. The dosages of radiation therapy were adjusted between 30 and 45 Gy, depending on each patient’s condition and tolerability.

### 2.3. Preoperative Evaluation and Decision-Making

In all cases, cervical LN metastases were primarily detected using FDG-PET/CT as hotspots in the neck. Ultrasound-guided fine-needle aspiration cytology or core needle biopsy was performed in all patients, and the pathology was confirmed to be consistent with the primary disease. Three patients with lung adenocarcinoma (cases 4, 6, and 7) underwent a core needle biopsy due to the need for genetic testing at the request of a medical oncologist.

Referring to the FDG-PET/CT images and the clinical characteristics of the patients, dissection for each case was decided in tumor board meetings held once every two weeks by the head and neck oncology team. In accordance with the treatment principles for clinical LN metastasis from head and neck cancer, all patients in this study underwent type II or type III modified radical ND. The ND classification proposed by the American Head and Neck Society and the American Academy of Otolaryngology-Head and Neck Surgery was used to classify the location of LNs [8].

### 2.4. Occult Metastasis Rate

To estimate the optimal ND level, we compared the pathologically positive and radiologically positive nodes detected using FDG-PET/CT preoperatively. FDG-PET/CT images were evaluated by a board-certified nuclear medicine physician and a head and neck surgeon. Subsequently, focal FDG uptake corresponding to the LNs identified on CT was counted as c+Ns. During ND, the contents of level Ia to level Vb specimens were dissected, labeled, and processed separately from the main specimen. Subsequently, the surgical specimens were sent to the pathology department for permanent section analysis. Histopathological examination of the metastases included identifying the number and location of nodes containing metastatic disease. In total, 583 resected nodes at 200 cervical LN levels underwent pathological evaluation. LNs with malignant nests were considered pathologically positive nodes (p+Ns). The occult metastasis rate of each cervical neck level was calculated by dividing the number of patients with histopathologically confirmed metastases at that level (but no preoperative evidence of such metastases) by the number of patients assessed (the patient’s relevant cervical neck level was assessed as clinically node-negative preoperatively) and was expressed as a percentage.

### 2.5. Lymph Node Ratio (LNR)

To further explore clinicopathologic factors impacting prognosis, we analyzed the LN ratio (LNR), which is defined as the ratio of positive LNs to the total number of LNs removed.

### 2.6. Statistical Analysis

Chi-square or Fisher’s exact tests were used to elucidate the clinicopathological factors affecting occult cervical LN metastasis. Survival analysis was performed using the Kaplan–Meier method. Survival rates were compared using the log-rank test between the two groups. Statistical Package for Social Sciences version 26 (IBM Corporation, Armonk, NY, USA) software was used for statistical analysis; *p* < 0.05 was considered statistically significant.

## 3. Results

### 3.1. Distribution of p+Ns according to Primary Site

The distribution of the dissected level and the level with p+N values is shown in Table 2.

Preoperatively, the most detected level of metastasis was level IV (inferior jugular chain) on the left side, followed by level four on the right side, and level Vb (lower posterior LN group) on the left side. The most pathological positive level was also level four on the left side, followed by level three on the left side. Metastases from the lungs, mammary glands, and upper GI tract occurred on both sides, whereas lesions in organs lower than the diaphragm (ovary and colorectal) tended to occur only on the left side.

### 3.2. Distribution of c+Ns, p+Ns, and Occult Nodal Metastasis according to the Cervical Neck Level

The distributions of c+Ns and p+Ns in each patient are listed in Table 3. Forty-five PET+Ns (c+Ns) were observed at 33 levels in 25 patients. However, 85 p+Ns (14.6%) occurred among 583 dissected nodes in 50 (25.0%) out of 200 levels. The distribution of p+N and c+N levels according to the neck level is shown in Table 2. In this table, the numerators indicate the number of c+N cases, and the denominators indicate the number of p+N cases at each level. 

**Figure 1:**
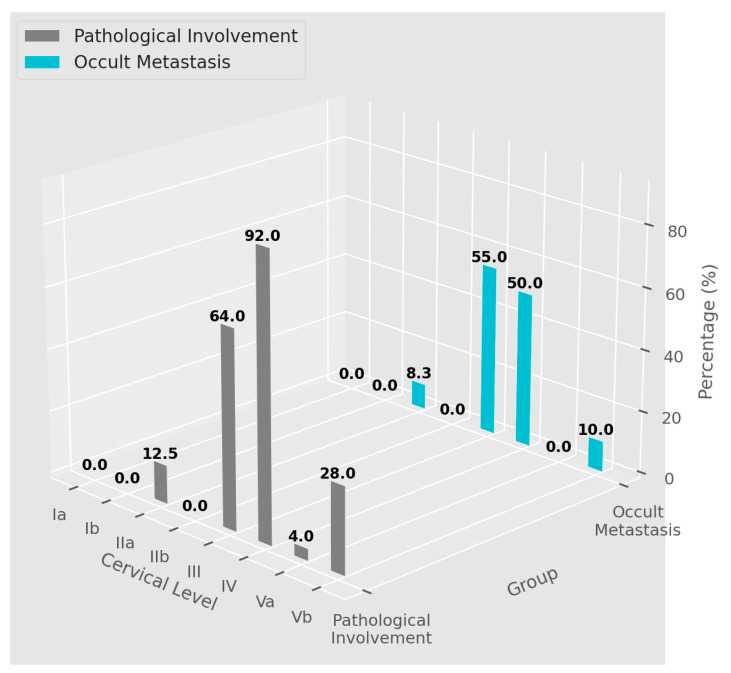
Distribution of Pathological and Occult LN Metastases by Cervical Level. The figure presents the percentage of pathological (gray) and occult (blue) LN metastases at cervical levels Ia through Vb. Pathological involvement is confirmed through histopathology after dissection, while occult metastasis is undetected clinically but identified post-surgery. Notably, pathological involvement peaks at level IV (92%), while occult metastasis is most prevalent at level III (55%). Levels Ia, IIb, and Va show no pathological involvement.

The ratios of LN metastasis and occult LN metastasis according to the cervical neck level are shown in Table 4 and Figure 1. Cervical level IV showed the highest rate of pathological LN metastasis at 92.0%, followed by level III at 64.0%. In contrast, level III presented the highest rate of occult metastasis at 64.0%, with level IV following at 50.0%. Postoperatively, at level IIa, three out of 25 patients (12.5%) showed LN metastasis; of these, two patients (one with a primary lung tumor and another with a mammary gland tumor) had occult metastasis not identified on preoperative imaging, yielding an occult metastasis rate of 8.3%. At level Va, only one of the 25 patients (4.0%, with a primary mammary gland tumor) exhibited pathologically confirmed LN metastasis, previously detected by preoperative PET-CT, indicating no occult metastasis. Additionally, in the subgroups of level I (Ia and Ib) and level IIb, no pathologically positive LNs or occult LN metastases were identified among the patients in this study.

### 3.3. Clinicopathologic Factors Affecting Occult Cervical LN Metastasis

We analyzed the relationship between occult LN metastasis and several clinicopathological factors in the 25 patients (Table 5). Although no statistically significant differences were observed in age, sex, primary site, number of levels involved, or LN size, univariate analysis indicated a higher likelihood of occult metastasis in cases where an increased number of involved LNs (*p* = 0.012) and extra-nodal extension (ENE) were present (*p* = 0.008). Multivariate logistic regression analysis identifies the number of involved LNs as a statistically significant predictor, with an odds ratio suggesting a notable increase in the likelihood of occult metastasis as the number of involved LNs increases (*p* = 0.031). Conversely, extra-nodal extension (ENE), while initially significant in univariate analysis, did not retain statistical significance in this multivariate context (Table 6).

### 3.4. Survival

The average follow-up period for the 18 patients was 45.6 ± 14.4 months, during which 13 patients experienced recurrence. The recurrence sites varied: six patients experienced recurrence in the lung/mediastinum, four in the neck, two in the abdomen, and one in the bone. Among these, two patients were successfully treated with radiotherapy and/or additional chemotherapy. Cancer was the cause of death in eleven cases.

Specifically, neck recurrences in four patients predominantly occurred in the ipsilateral supraclavicular fossa, just behind the clavicle (cases 6 and 9 with lung cancer and case 14 with ovarian cancer). One patient with esophageal cancer exhibited recurrence at the contralateral level IV of the neck.

**Figure 2:**
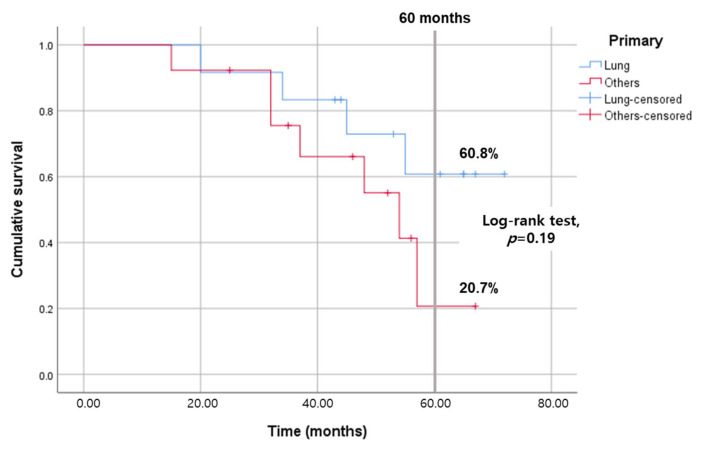
Comparison of disease-specific survival between the lung cancer group and other cancer groups using the Kaplan–Meier method. The log-rank test was employed to determine statistical significance. No significant differences were observed between the two groups (*p* = 0.19).

**Figure 3:**
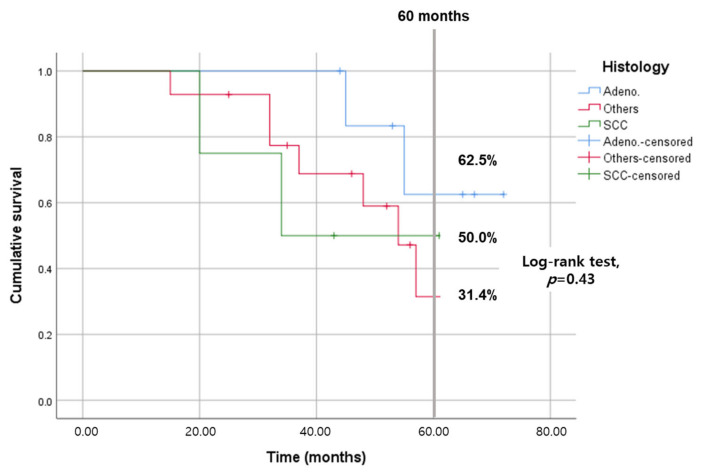
Kaplan–Meier survival analysis comparing disease-specific survival among groups with adenocarcinoma, squamous cell carcinoma (SCC), and other histological types. The log-rank test indicated no significant survival differences between the groups (*p* = 0.43).

The five-year disease-specific survival (DSS) rate for the entire cohort was 44.3%. Patients with lung cancer demonstrated a relatively higher DSS rate (60.8%) compared to those with other types of malignancies (20.7%), although this difference was not statistically significant (*p* = 0.29), as illustrated in Figure 2. DSS rates varied across pathological groups, including 62.5% for adenocarcinoma, 50.0% for squamous cell carcinoma, and 31.4% for other histological types, as shown in Figure 3. 

**Figure 4:**
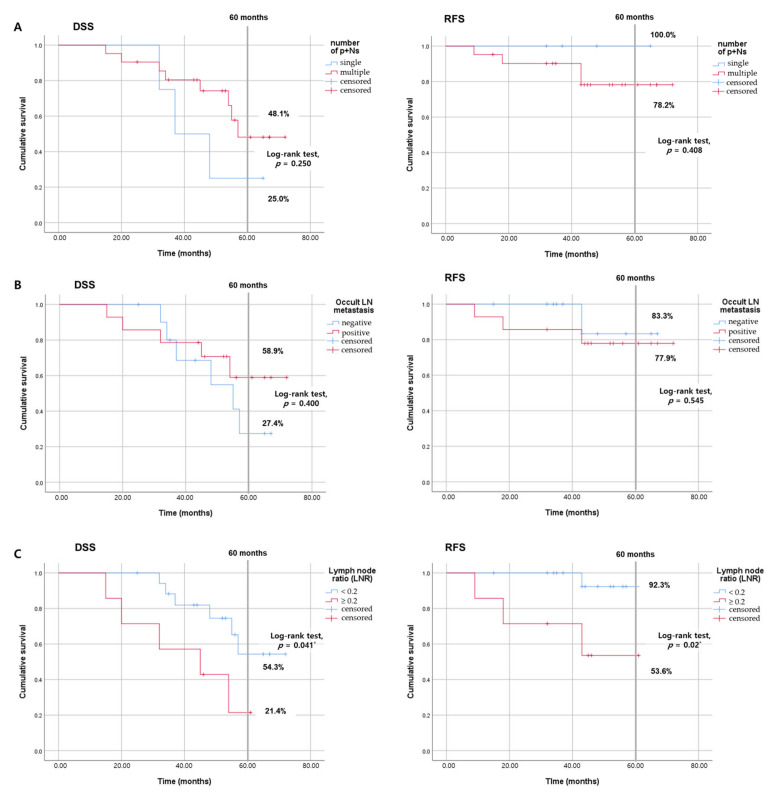
Kaplan–Meier analyses of disease-specific (DSS) and recurrence-free survival (RFS) across different prognostic factors. (**A**) For the number of p+N, no significant differences in DSS (*p* = 0.250) or RFS (*p* = 0.408) were observed between patients with n = 1 and those with n ≥ 2. (**B**) Analysis of occult lymph node (LN) metastasis showed similar DSS (*p* = 0.400) and RFS (*p* = 0.545) for patients with and without occult LN metastasis. (**C**) However, when stratified using a lymph node ratio (LNR) of 0.2, significant disparities emerged, with improved DSS (*p* = 0.041) and RFS (*p* = 0.02) in patients below the cutoff, highlighting the prognostic value of LNR. *, *p* < 0.05.

The number of pathologically positive nodes ranged from 1 to 6 (mean ± SD = 3.4 ± 1.5). When categorized into single or multiple according to the number of pathologically positive LN, four patients had single, and 21 had multiple p+Ns. The five-year DSS and recurrence-free survival (RFS) rates were 25.0% and 100.0% for single p+N group, and 48.1% and 78.2% for the multiple p+Ns group, respectively (*p* = 0.250 and 0.408, Figure 4A). Of the 25 patients, 14 had occult metastases. The five-year DSS and RFS rates for patients without occult metastasis were 27.4% and 83.0%, whereas these rates were 58.9% and 77.9% (*p* = 0.400 and 0.545), respectively, for those with occult metastasis, as depicted in Figure 4B.

Recognized not only for its association with prognosis in various cancers, including head and neck cancer, the LNR has also proven superior to conventional LN staging in predicting survival outcomes [9]. We divided the patients into two groups based on their LNR: ≥0.2 and <0.2. Eighteen patients were in the LNR < 0.2 group and 7 were in the LNR ≥0.2 group. As demonstrated in Figure 4C, patients with an LNR ≥ 0.2 exhibited poorer five-year DSS (21.4% vs. 54.3%, *p* = 0.041) and RFS (53.6% vs. 92.3%, *p* = 0.020).

## 4. Discussion

This study aimed to explore the possibility of limiting the extent of ND by safely excluding certain neck levels in cases with cervical LN metastases originating from remote primary malignancies. As recent cutting-edge research has accumulated data, it is increasingly evident that surgically removing cervical LN metastasis from remote primary tumors can significantly reduce tumor burden and potentially improve survival rates [1,3,10]. This underscores the critical role of the surgical management of neck diseases as a diagnostic and therapeutic strategy for treating these patients. However, each case must be evaluated individually, considering the associated primary tumor, resectability, and comorbidities [1]. 

Previous studies have provided insights into the patterns of cervical LN metastasis at various remote primary sites. Breast cancer has been identified as the most frequent distant primary site metastasis to the cervical neck, with a reported prevalence of 2.3–4.3%. In these cases, supraclavicular LNs are most affected, whereas metastases to the jugular chain are less common [1]. Additionally, Davis et al. reported a wide-ranging incidence (1.5–32%) of neck node metastasis from primary lung cancer, which could partly be attributed to the non-specific use of the term ‘cervical node’ [11]. Similarly, Nagarkar et al. observed that 41.17% of cervical LN metastases originate from lung cancer as the primary malignancy. This was followed by 20.57% of primary malignancies in the head and neck (excluding the oral cavity) and 17.64% of breast cancers [12]. These findings are consistent with our results, which also identified lung cancer as the most common remote primary source of cervical LN metastases. This consistency across studies underscores the significance of lung cancer in cervical LN involvement.

In our study, we observed a distinctive pattern of cervical LN involvement in metastasis. Although the overall prevalence of LN metastasis was higher on the left side than on the right side, consistent with previous reports, the specific levels of involvement differed from previously reported patterns. Prior studies have indicated that the most frequently affected sites are the left supraclavicular LNs (SCLN), followed by the right SCLN, and then levels IV, II, and V, in descending order [12]. In contrast, our data revealed that the left level IV was the most involved site, followed by the left level III, right level IV, left level Vb (including the SCLN group), right level III, right level Vb (including the SCLN group), and left level IIa. Interestingly, while pathological LN involvement was most frequent at level IV, occult metastasis occurred predominantly at level III (55.0%), followed by level IV (50.0%), level II (8.3%), and level Vb (10.0%), with no occult metastasis at levels I and Va. Given the standard threshold (10–20%) for performing prophylactic ND, levels III, IV, and Vb should be included in every dissection for cervical LN metastasis from the remote primary site to achieve therapeutic intent [13]. Although these findings do not allow us to draw definitive conclusions, they do offer preliminary clinical insights. In cases of HNSCC with c+N, the standard practice of performing mRND to cover all cervical levels may not be necessary, particularly for cervical LN metastases from remote primaries. Our findings suggest that if clinical staging does not indicate upper neck LN metastasis, a more conservative approach focusing on levels III, IV, and Vb, which are most frequently associated with pathological and occult metastasis, could be optimal. This strategy may allow preservation of the upper cervical levels, potentially reducing morbidity while still addressing the most likely sites of metastatic involvement.

Additionally, pathological involvement of the left level IIa was observed in 3 out of 25 patients, corresponding to a rate of 12.5%. This incidence was higher than the rate of 7.35% reported in previous studies [12]. However, when analyzing the involvement of the upper cervical neck (levels I and II) as a whole, a different picture emerges. In our cohort, level I (0%) showed no involvement, whereas previous studies reported a 4.41% involvement rate at this level [12]. Consequently, the overall involvement rate of the upper cervical neck in our study was 12.5%, closely aligning with the rate of 11.76% reported in prior studies. Notably, in our cohort, level IIa occult metastasis was detected in two patients: one with lung cancer and the other with breast cancer. While the small sample size precluded a detailed analysis of the risk factors for occult metastasis at level IIa, it is intriguing that both patients had preoperative involvement at level III. Although further investigation with a larger cohort is required for a robust analysis, these preliminary findings suggest that expanding the extent of dissection to level IIa may be considered for patients with lung and breast cancer with preoperative level III involvement.

We also assessed the clinicopathological factors related to occult LN metastasis and found that the likelihood of occult metastasis was high when the number of involved LNs was higher and ENE was present. Given that the not only N stage but also the number of LNs metastasis can help predict the risk of LN metastasis in various types of cancer, these findings are significant [14,15,16,17]. For example, in several studies on non-small cell lung cancer, the number of LN metastasis was identified as a significant risk factor for poor outcome and occult LN metastasis [15,16,17]. Similarly, in a study on oral cavity cancer, the N stage was identified as a predictor of occult LN metastasis at cervical level IV, with the study concluding that occult metastasis is more prevalent in cases of N2b or higher compared to N1 or N2a. This indirectly suggests that the number of LN metastases is an important factor in determining the likelihood of occult metastasis [18]. Additionally, while ENE was initially identified as a significant factor correlating with occult metastasis in univariate analysis, its significance was diminished in a multivariate analysis. This finding necessitates a nuanced interpretation of ENE’s clinical implications regarding occult metastasis. ENE, characterized by the spread of metastatic cancer cells beyond the lymph node capsule into adjacent soft tissue, is a well-established poor prognostic factor and a documented risk factor for occult metastasis in various cancers [19,20,21]. Despite its reduced statistical significance in multivariate analysis, the potential for microscopic lymph node extension or occult lymph node metastasis associated with ENE cannot be completely disregarded. This understanding underscores the importance of vigilant evaluation and management strategies for patients with ENE. Their condition may significantly influence therapeutic decisions and prognostic outcomes, necessitating further research, preferably with larger cohorts, to elucidate the role of ENE in occult metastasis and guide more effective clinical interventions.

Moreover, in our assessment of patients with lung cancer, the 5-year DSS rate was 60.8% and even higher at 62.5% for those with adenocarcinoma, despite all patients being categorized as stage IIIB or above. This finding is encouraging, especially considering that the National Cancer Institute reports the 5-year survival rate for stage IIIB lung cancer to be approximately 25% [22]. This improved outcome may be attributed not only to recent advancements in anticancer agents, which are increasingly based on genetic and immunological precision in the field of lung cancer, but also to enthusiastic efforts to surgically reduce the tumor burden [23]. However, in our study, no significant differences in survival or recurrence were identified according to nodal stage or occult metastasis. This likely results from the fact that all patients uniformly underwent mRND, ensuring a comprehensive surgical scope. Consequently, the likelihood of residual microscopic or occult metastasis postoperatively is reduced. 

The LNR was significantly correlated with DSS and RFS. This aligns with the recent literature positing the LNR as a superior prognostic indicator compared to nodal staging in various cancers, including head and neck cancers [9,24,25,26,27]. The LNR reflects not only the LN metastatic burden but also the thoroughness of lymphadenectomy. Ding et al. identified the LNR as an independent predictor for overall survival and DSS in oral squamous cell carcinoma, outperforming the AJCC on Cancer TNM classification. A high LNR indicates a 2.5-fold increase in the risk for mortality and recurrence [28]. Similarly, Soran et al. found that while both the LNR and nodal staging effectively predicted OS in breast cancer, the LNR was more indicative of the risk of distant recurrence. Specifically, patients with a triple-negative phenotype and an LNR > 0.2 faced double the mortality risk compared to those with an LNR < 0.2 [25]. Chiappetta et al. also recognized the LNR as an independent factor for OS and disease-free survival in patients with NSCLC undergoing curative resection [29]. These findings corroborate our results, particularly regarding DSS and RFS, in patients with an LNR > 0.2.

This study, while shedding light on the surgical management of cervical LN metastases from remote primary tumors, has limitations that merit further exploration. First, its retrospective nature raises questions about data reliability, and the small patient cohorts limit the generalizability of the findings. Second, the absence of a comparative analysis between different ND types (mRND and selective ND (levels III, IV, and Vb)) hinders a more nuanced understanding of recurrence and oncologic outcomes. Furthermore, the limited sample size, compounded by the heterogeneity of the primary site and adjuvant chemoradiation therapy, restricts the exploration of clinicopathological parameters related to occult metastasis, particularly at neck levels IIa and Vb. One major advantage of this study was that our patients underwent a uniform extent of neck surgery for mRND at a single institution. While offering valuable insights into the management of cervical LN metastases from distant primary tumors, the retrospective nature and limited sample size, particularly in certain subgroups, warrant further prospective research. More comprehensive comparisons between different ND methods are needed to enhance our understanding and treatment strategies in this complex and heterogeneous clinical scenario.

## 5. Conclusions

Our results recommend including levels III, IV, and VB in therapeutic NDs for cervical LN metastases from remote primaries, while suggesting that the upper ND might be omitted. However, larger prospective and comparative studies are required to confirm the optimal extent of surgery.

## Figures and Tables

**Table 1:** Patients’ characteristics.

Case	Age	Sex	Primary	Histology	Previous Treatment			ND Side	Adjuvant Therapy	
					Surgery	CT	RT		CT	RT
1	54	M	Lung	Adeno.		Y	Y	L	Y	
2	49	F	Lung	Adeno.		Y	Y	L	Y	
3	61	M	Lung	Adeno.		Y	Y	L	Y	
4	62	F	Lung	Adeno.		Y		R		
5	55	M	Lung	Adeno.		Y	Y	L	Y	
6	41	M	Lung	Adeno.		Y	Y	L	Y	
7	49	F	Lung	Adeno.	Y	Y	Y	R	Y	
8	66	M	Lung	Adeno-squamous		Y	Y	L	Y	
9	56	F	Lung	SCC		Y	Y	L	Y	Y
10	60	M	Lung	SCC	Y	Y	Y	R	Y	Y
11	70	M	Lung	SCC	Y	Y	Y	L	Y	
12	72	M	Lung	Pleomorphic carcinoma		Y	Y	L		
13	59	F	Ovary	Endometrioid adeno.	Y	Y	Y	L	Y	
14	67	F	Ovary	Endometrioid adeno.	Y	Y	Y	L	Y	
15	54	F	Ovary	Endometrioid adeno.	Y	Y		L	Y	
16	66	F	Ovary	Endometrioid adeno.	Y	Y		L	Y	
17	68	F	Mammary	Adeno.	Y	Y	Y	R	Y	Y
18	47	F	Mammary	Adeno.	Y	Y	Y	L	Y	Y
19	40	F	Mammary	Adeno.	Y	Y	Y	L	Y	Y
20	73	M	Esophagus	Adeno.	Y	Y		R		Y
21	60	F	Esophagus	SCC	Y	Y	Y	L	Y	Y
22	54	M	Esophagus	SCC	Y	Y		L		Y
23	40	M	Thymus	Malignant thymoma	Y	Y	Y	R		
24	74	M	Thymus	Malignant thymoma	Y	Y		L		
25	48	M	Colon	Adeno.	Y	Y	Y	L	Y	

Adeno., adenocarcinoma; SCC, squamous cell carcinoma; CT, chemotherapy; RT, radiotherapy; L, left; R, Right.

**Table 2:** Distribution of c+Ns and p+Ns according to the cervical neck level.

	Lung	Ovary	Mammary	Esophagus	Thymus	Colon	Total
L1a			0/0	0/0	0/0	0/0	0/0
L1b			0/0	0/0	0/0	0/0	0/0
L2a	1/2	0/0	0/1	0/0	0/0	0/0	1/3
L2b	0/0	0/0	0/0	0/0	0/0	0/0	0/0
L3	1/6	0/4	1/1	0/0	1/1	0/0	3/12
L4	7/9	4/4	1/1	2/2	0/0	1/1	15/17
L5a	0/0	0/0	1/1	0/0	0/0	0/0	1/1
L5b	2/2	1/1	0/0	1/1	0/0	0/0	4/4
R1a			0/0	0/0	0/0		0/0
R1b			0/0	0/0	0/0		0/0
R2a	0/0		0/0	0/0	0/0		0/2
R2b	0/0		0/0	0/0	0/0		0/0
R3	1/2		0/0	0/1	1/1		2/4
R4	3/3		1/1	1/1	1/1		6/6
R5a	0/0		0/0	0/0	0/0		0/0
R5b	1/3		0/0	0/0	0/0		1/3
Total	16/27	5/9	4/5	3/4	3/3	1/1	33/52

L, left; R, right; c+Ns, clinically positive nodes; p+Ns, pathologically positive nodes; numerators indicate the number of c+Ns cases and denominators indicate the number of p+Ns cases in each level.

**Table 3:** Clinicopathologic features of dissected LNs including the distribution of c+Ns, p+Ns, and occult nodal metastasis, categorized according to the cervical neck levels.

Case	Total Harvested LN	Distribution of c+N/p+N	ENE	Maximal Size of p+LN (cm)	Number of LNs		Number of Levels		Occult Nodal Metastasis	
					c+Ns	p+Ns	c+Ns	p+Ns		Level of Metastasis
1	38	IV/III, IV	+	2.30	2	5	1	2	Y	III
2	30	IV/III, IV	+	1.40	2	5	1	2	Y	III
3	33	IIa, IV/IIa, III, IV	+	2.60	2	5	2	3	Y	III
4	29	IV/III, IV, Vb	−	1.90	1	4	1	3	Y	III, Vb
5	31	III, IV/IIa, III, IV	−	1.50	2	3	2	3	Y	IIa
6	25	Vb/Vb	+	2.40	1	4	1	1		
7	19	IV/IV, Vb	−	2.70	2	4	1	2	Y	Vb
8	28	Vb/Vb	−	3.90	1	4	1	1		
9	27	IV/III, IV	+	2.70	3	6	1	2	Y	III
10	15	III, IV, Vb/III, IV, Vb	−	1.80	3	3	3	3		
11	19	IV/III, IV	+	1.30	1	4	1	2	Y	III
12	15	IV/IV	−	1.30	1	1				
13	14	IV/III, IV	−	2.90	1	3	1	2	Y	III
14	22	IV/III, IV	+	1.21	2	5	1	2	Y	III
15	34	IV/III, IV	−	1.50	2	5	1	2	Y	III
16	32	IV, Vb/III, IV, Vb	−	1.70	2	5	2	3	Y	III
17	24	IV/IV	−	1.70	1	2	1	1		
18	19	III, Va/IIa, III, Va	+	3.10	3	4	2	3	Y	IIa
19	23	IV/IV	−	1.50	1	1	1	1		
20	14	IV/III, IV	−	3.70	2	3	1	2	Y	III
21	10	IV, Vb/IV, Vb	−	1.30	1	2	2	2		
22	15	IV/IV	−	0.80	2	2	1	1		
23	29	III, IV/III, IV	−	2.10	2	3	2	2		
24	15	III/III	−	0.90	1	1	1	1		
25	23	IV/IV	−	1.10	1	1	1	1		
mean	23.32 ± 7.61				1.97 ± 0.84	1.68 ± 0.69	3.40 ± 1.50	1.33 ± 0.56		

c+Ns, clinically positive nodes; p+Ns, pathologically positive nodes; ENE, extra-nodal extension; LN, lymph node; +, positive for ENE; −, negative for ENE.

**Table 4:** The ratio of LN metastasis involvement and occult LN metastasis based on cervical neck levels.

Cervical Level	Pathological Involvement of LN Metastasis (%)	Occult LN Metastasis (%)
Ia	0	0
Ib	0	0
IIa	12.5% (3/25)	8.3% (2/24)
IIb	0	0
III	64.0% (16/25)	55.0% (11/20)
IV	92.0% (23/25)	50.0% (2/4)
Va	4.0% (1/25)	0
Vb	28.0% (7/25)	10.0% (2/20)

**Table 5:** Clinicopathologic factors affecting occult cervical LN metastasis.

Variables	Patients with Occult LN Metastasis, No. (%)	*p*-Value
Age (years)		0.656
<50	3/7 (42.9%)	
≥50	11/18 (61.1%)	
Gender		0.111
Male	5/13 (38.5%)	
Female	9/12 (75.0%)	
Primary		0.119
Lung	8/12 (66.7%)	
Ovary	4/4 (100%)	
Mammary	1/3 (33.3%)	
Esophagus	1/3 (33.3%)	
Thymus	0/2 (0%))	
Colon	0/1 (0%)	
Number of involved levels		0.649
1	10/18 (55.6%)	
≥2	4/7 (57.1%)	
LN size		0.414
≤2 cm	7/15 (46.7%)	
>2 cm	7/10 (70.0%)	
Number of involved LNs		0.012 *
1	0/4 (0.0%)	
2	0/3 (0.0%)	
3	3/5 (60.0%)	
4	4/6 (66.7%)	
5	6/6 (100.0%)	
6	1/1 (100.0%)	
Extra-nodal extension		0.008 **
Negative	7/18 (38.9%)	
Positive	7/7 (100%)	

*, *p* < 0.05; **, *p* < 0.01.

**Table 6:** Multivariate logistic regression model for affecting occult cervical LN metastasis.

Variables	B	S.E.	OR	95% CI	*p*-Value
Number of involved LNs	2.125	0.983	8.375	(1.219~57.520)	0.031 *
Extra-nodal extension	0.873	1.776	2.393	(0.074~77.780)	0.623
−2*LL* = 16.792, NagelKerke R^2^ = 0.675, Hosmer and Lemeshow test: χ^2^ = 2.164 (*p* = 0.826)					

*, *p* < 0.05; S.E., standard error; OR, odds ratio; CI, confidence interval.

## Data Availability

The data presented in this study are available on request from the corresponding author. The data are not publicly available due to privacy or research ethics.
